# A Unique Case of Alpha-Fetoprotein-Negative Hepatoid Adenocarcinoma of the Stomach With Associated Signet Ring Cell Histological Features and Linitis Plastica

**DOI:** 10.7759/cureus.11908

**Published:** 2020-12-04

**Authors:** Thomas A Wichelmann, Komal Patel, Abdullah Malas, Edward James

**Affiliations:** 1 Internal Medicine, Advocate Lutheran General Hospital, Park Ridge, USA; 2 Oncology, Advocate Lutheran General Hospital, Park Ridge, USA

**Keywords:** hepatoid adenocarcinoma, stomach, metastatic stomach cancer, gastric cancer

## Abstract

Hepatoid adenocarcinoma of the stomach (HAS), a rare and unique histological subtype of gastric cancer, accounts for less than 1.5% of all gastric cancers. Historically, this subtype is found to have a poor prognosis in comparison to other types of gastric cancer. While the diagnosis is made based on pathological findings, most cases described in the literature are associated with elevated alpha-fetoprotein (AFP) levels. We present a case of AFP-negative HAS with additional unique pathologic findings of signet ring cells which has been reported only once in the literature. Given the rare and late presentation of the disease, AFP-negative HAS should be included in the differential diagnosis in patients with suspicion for gastric cancer.

## Introduction

Hepatoid adenocarcinoma of the stomach (HAS) represents a rare and unique histological subtype of gastric cancer that has been associated with liver metastasis and poor prognosis. It accounts for 1.3-1.5% of gastric cancers and occurs most commonly in middle-aged males [1]. Hepatoid adenocarcinoma is most often identified in the stomach; however, it can be found in various other organs or tissues such as the lungs, esophagus, gallbladder, pancreas, papilla of Vater, colon, jejunum, rectum, peritoneum, renal pelvis, ureter, bladder, ovaries and uterus [1].

Serum alpha-fetoprotein (AFP) is a plasma protein that is most commonly used as a tumor marker and independent risk predictor for hepatocellular carcinoma (HCC) [2]. While several immunohistochemical markers have been identified in association with HAS (AFP being the most common marker, along with glypican 3 [GPC-3], Sal-like 4 [SALL4], and Hep-Par 1), the diagnosis of HAS is made by several histopathological characteristics resembling hepatocellular carcinoma (HCC). As described by Nagai et al, HAS is histologically defined by “polygonal tumor cells with either abundant eosinophilic or a clearly granular cytoplasm proliferating in a solid or trabecular fashion, resembling hepatocellular carcinoma”[3]. While most cases of HAS described in the literature are associated with AFP positivity, the diagnosis of HAS is defined based on morphological patterns of hepatocyte differentiation on biopsy as confirmed in prior evaluation by Supriatna et al., confirming albumin messenger RNA (mRNA) presence in AFP-negative gastric adenocarcinoma with hepatoid morphological features [4].

## Case presentation

A 62-year-old Polish male with no significant past medical history presented with a two-month history of epigastric and umbilical abdominal pain. He described additional abdominal distension and decreased appetite with a 30 lb weight loss over three months. He denied any associated symptoms of nausea, vomiting, melena, or hematochezia. No prior esophagogastroduodenoscopy (EGD) or colonoscopy history. The patient was a former smoker who had quit 4.5 years ago. He reported infrequent alcohol use. 

**Figure 1:**
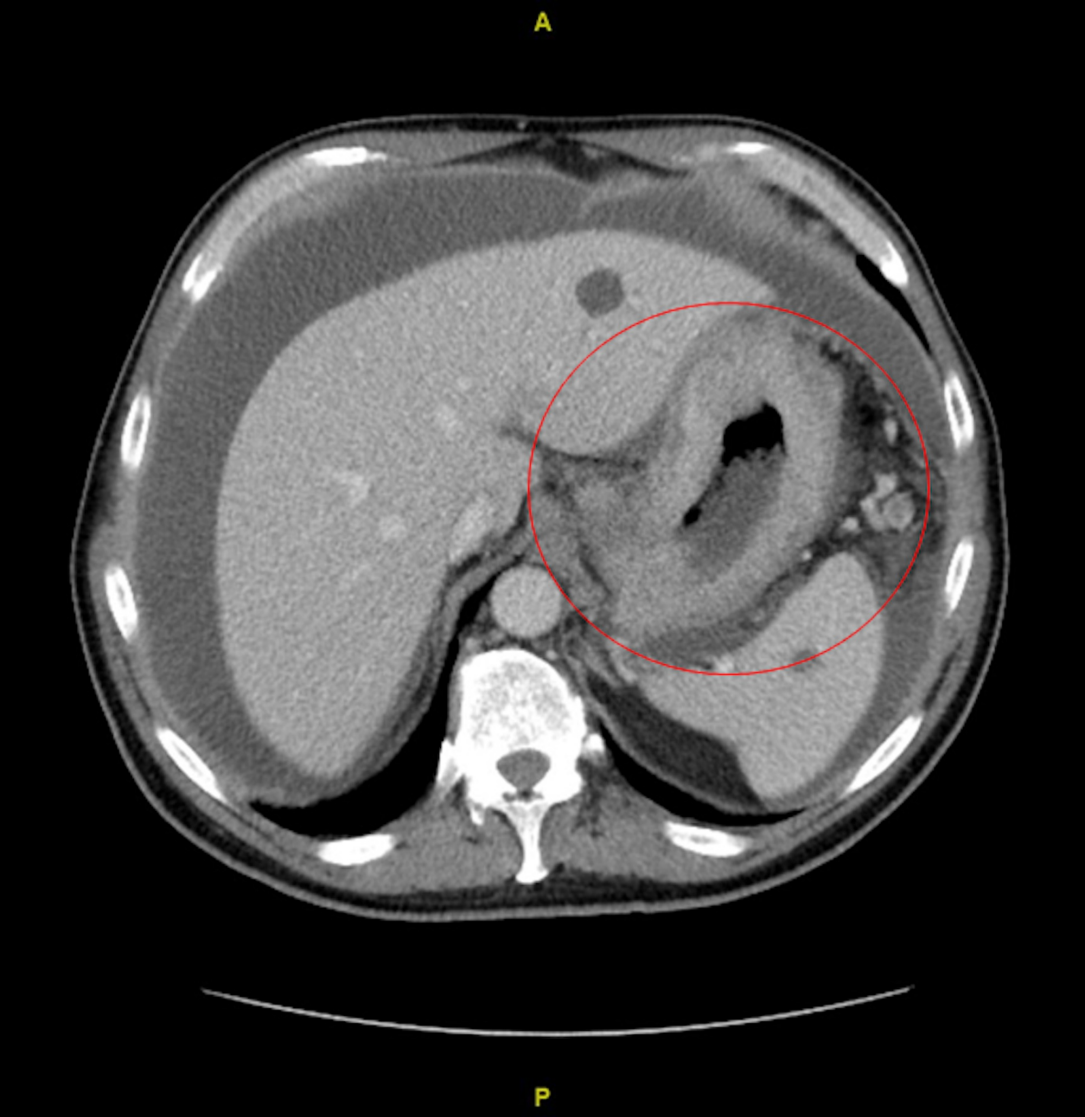
CT Abdomen and Pelvis (axial view) demonstrating gastric wall thickening and significant ascites. Incidental cysts can be visualized in the liver and spleen.

**Figure 2:**
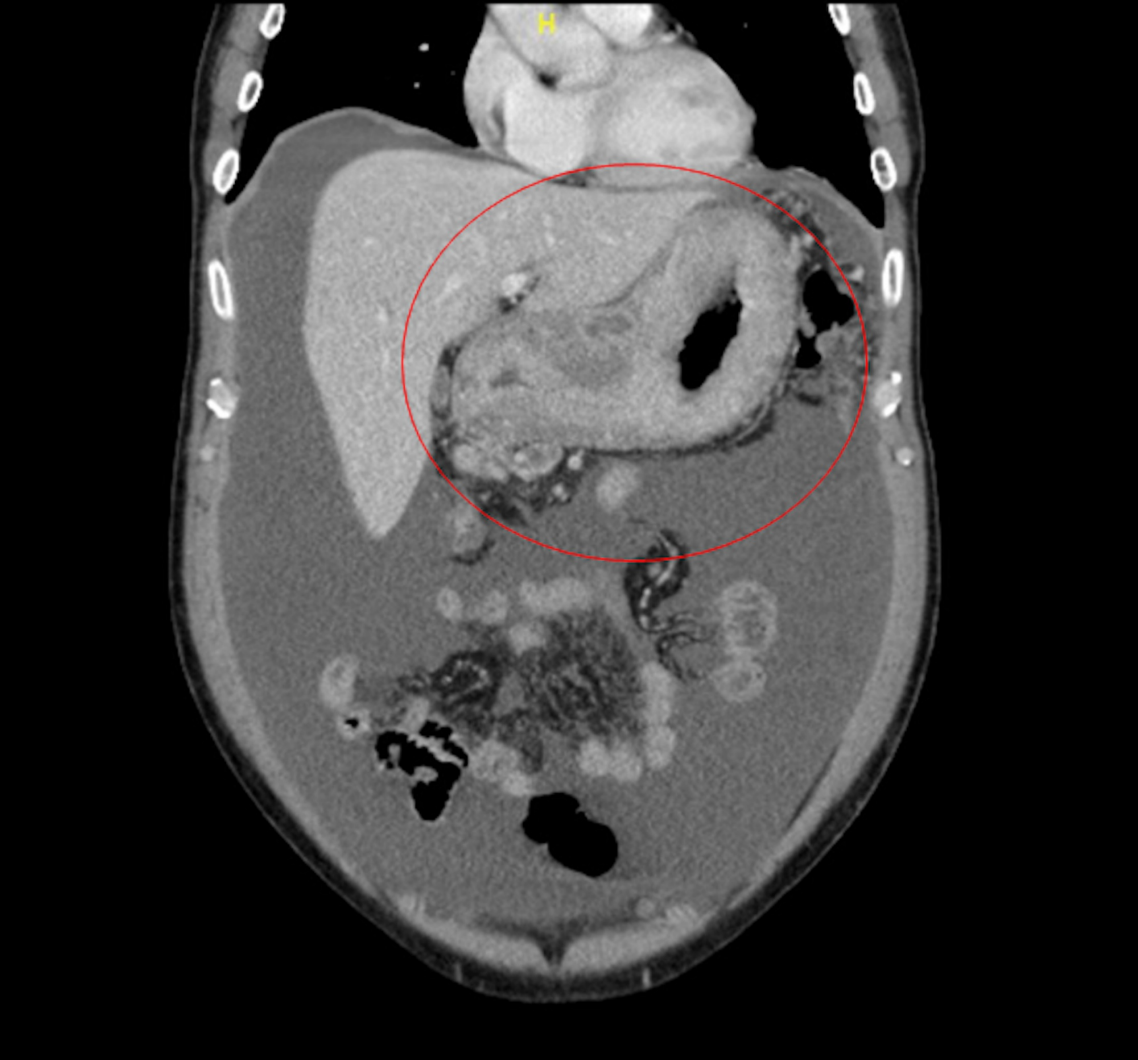
CT Abdomen and Pelvis (coronal view) demonstrating significant gastric wall thickening and ascites.

In the emergency room, vital signs were stable. Physical exam was significant for mild epigastric tenderness and moderate abdominal distension, but was otherwise unrevealing. Labs were remarkable for lipase 285 Units/L, albumin 2.3 g/dL, hemoglobin 11.0 g/dL with normal mean corpuscular volume (MCV), platelets 339 K/mcL and carcinoembryonic antigen (CEA) 3.6 ng/mL. Basic metabolic panel (BMP) was unremarkable. Computerized tomography (CT) abdomen and pelvis revealed a large tumor in the abdomen and pelvis with gastric wall thickening and extension into the soft tissues, large lymph nodes in the gastric hepatic ligament, peritoneal thickening, ascites, and omental caking favoring primary gastric cancer with peritoneal carcinomatosis (Figures 1, 2). Several small pulmonary nodules were noted. No additional metastatic disease was identified.

**Figure 3:**
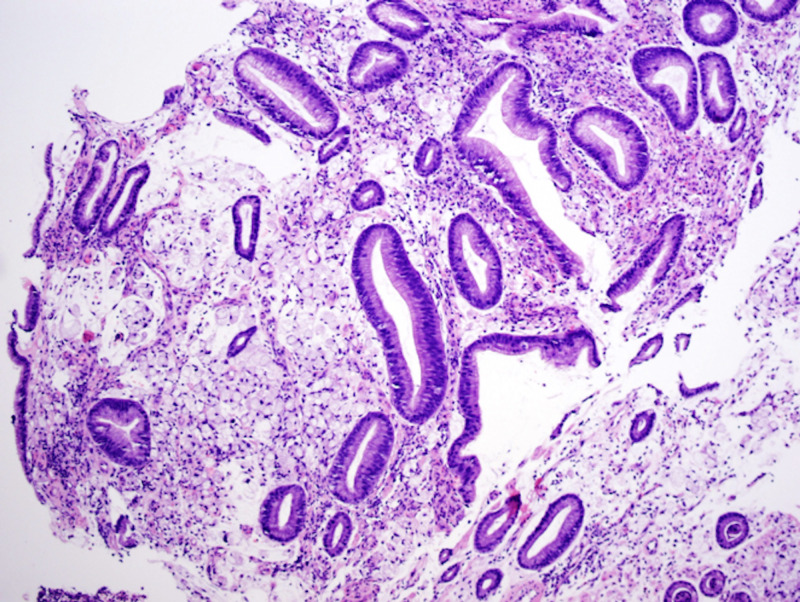
Gastric biopsy H&E staining, 10x, showing signet ring cells. H&E: Hematoxylin and eosin

**Figure 4:**
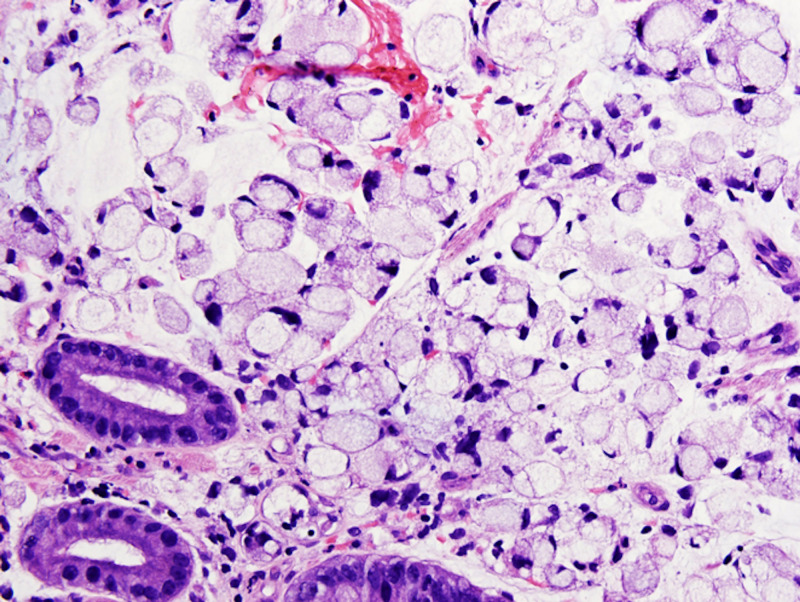
Gastric biopsy H&E staining, 40x, showing signet ring cells. H&E: Hematoxylin and eosin

**Figure 5:**
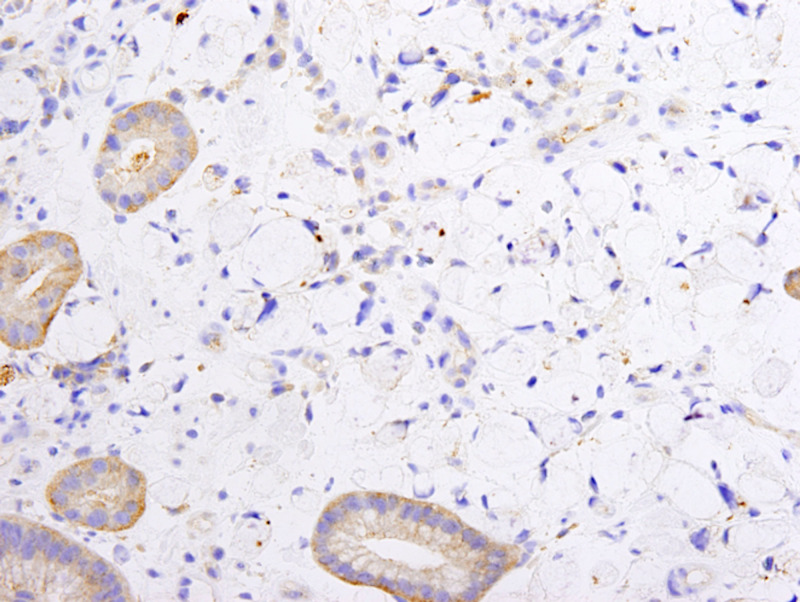
Gastric biopsy HepPar staining, 40x, with signet ring cells.

**Figure 6:**
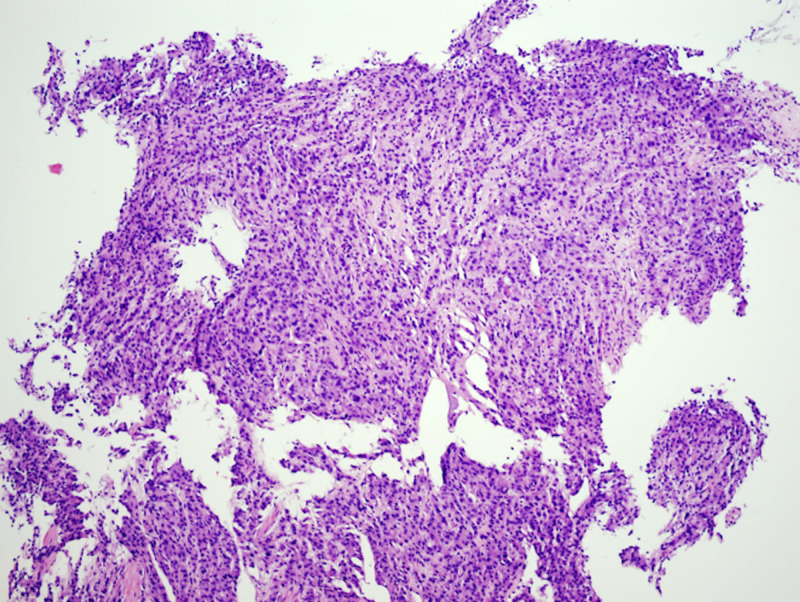
Gastric biopsy H&E staining, 10x, showing cells with hepatoid morphology. H&E: Hematoxylin and eosin

**Figure 7:**
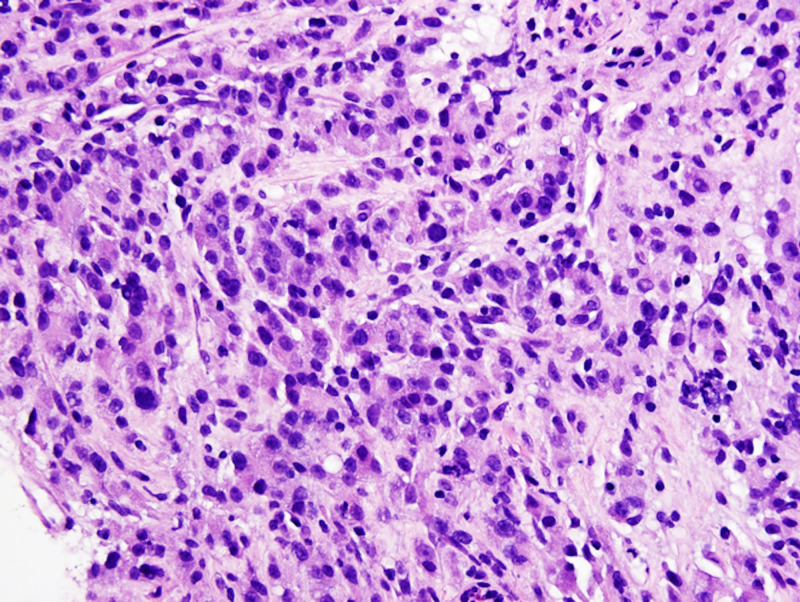
Gastric biopsy H&E staining, 40x, showing cells with hepatoid morphology. H&E: Hematoxylin and eosin

EGD revealed a large non-bleeding, circumferential mass occupying the entire stomach. Biopsy was consistent with combined features of signet ring cell carcinoma (linitis-plastica type) and hepatoid adenocarcinoma (HepPar1+) with microsatellite stability (MLH1, MSH2, PMS2, and MSH6) (Figures 3-7). Immunostains for AFP and human epidermal growth factor receptor 2 (HER-2/neu) were negative. Subsequent serum AFP was not elevated at 2.0 ng/mL.

The patient required large-volume paracentesis for malignant ascites and was started on folinic acid (leucovorin), fluorouracil (5-FU), and oxaliplatin (FOLFOX) therapy. Follow up CT scan after four doses of FOLFOX chemotherapy showed that the gastric wall thickening and malignancy was similar to prior, however metastatic disease along the lesser curvature of the stomach improved. The left upper quadrant/omental peritoneal carcinomatosis was not significantly changed and few small pulmonary nodules were unchanged. Ascites volume decreased and the patient has not needed any further paracentesis. The patient has felt well throughout chemotherapy and has continued to work full time. Programmed death-ligand 1 (PDL1) testing has indicated a combined positive score (CPS) of 10, indicating that the patient may be a candidate for immunotherapy in the future at progression.

## Discussion

HAS is a rare and aggressive form of gastric cancer that is characterized as adenocarcinoma with hepatocyte differentiation as previously described. While most cases of HAS described in the literature are AFP-producing (positive with biopsy staining and in serum), the largest series to date on HAS by Nagai et al. described 13 cases of AFP-negative stained HAS and a literature review completed by Roberts et al. found positive serum AFP in 34 of 38 reviewed cases of HAS [3,5]. Statistical analysis has revealed no significant difference between the AFP-positive and AFP-negative groups with regard to survival rate, though it should be noted that HAS-type gastric cancer as a whole has represented an even poorer prognosis in comparison to other gastric cancers without hepatoid features [3]. In the largest case series to date by Nagai et al., HAS cases had an 11.9% five-year survival rate in comparison to a 38.2% five-year survival rate amongst non-hepatoid adenocarcinomas of the stomach in the study. There was no statistically significant difference in subgroup analysis between AFP-negative HAS and AFP-positive HAS survival rate in the study [3]. This particular case of gastric cancer illustrates a unique pathological finding of AFP-negative HAS associated with signet ring cell histological features. A thorough literature review reveals only one prior case with similar pathological features (AFP-negative HAS associated with signet ring cell morphology and linitis plastica) as described by Liu et al., [6] making this case a unique histological variant warranting description in the setting of poor prognosis conferred by HAS-type cancers [5].

## Conclusions

HAS is characterized as a unique type of gastric cancer with both adenoid and hepatocyte features. Although AFP expression is commonly found in this type of cancer, there have been few reported cases of AFP-negative HAS. This case report illustrates an unusual presentation of AFP-negative HAS with associated signet ring cell histological features and further reaffirms that clinicians should include HAS on their differential diagnosis in patients who are diagnosed with AFP-negative gastric cancer.
